# Effect of the KCa3.1 blocker, senicapoc, on cerebral edema and cardiovascular function after cardiac arrest — A randomized experimental rat study

**DOI:** 10.1016/j.resplu.2021.100111

**Published:** 2021-04-02

**Authors:** Frederik Boe Hansen, Niels Secher, Thomas Mattson, Bo Løfgren, Ulf Simonsen, Asger Granfeldt

**Affiliations:** a Department of Biomedicine, Aarhus University, Ole Worms Allé 4, 8000 Aarhus, Denmark; b Department of Clinical Medicine, Aarhus University, Palle Juul-Jensens Blvd. 82, 8200 Aarhus N, Denmark; c Department of Anesthesiology and Intensive Care, Aarhus University Hospital, Palle Juul-Jensens Blvd. 99, 8200 Aarhus N, Denmark; d Department of Internal Medicine, Randers Regional Hospital, Skovlyvej 15, 8930 Randers NE, Denmark; e Research Center for Emergency Medicine, Aarhus University Hospital, Palle Juul-Jensens Blvd. 161, 8200 Aarhus N, Denmark

**Keywords:** KCa3.1 channel, intermediate calcium-activated potassium channel, EDH, endothelial derived hyperpolarization factor, KCa2.x channels, small calcium-activated potassium channels, NSE, neuron specific enolase, SKA-31, naphtho[1,2-d]thiazol-2-ylamine, FFM, mix of fentanyl, fluanisone and midazolam, FS, fractional shortening, Cardiac arrest, Senicapoc, KCa3.1, Cerebral edema, Cardiovascular, Rat model

## Abstract

**Aim:**

Formation of cerebral edema and cardiovascular dysfunction may worsen brain injury following cardiac arrest. We hypothesized that administration of the intermediate calcium-activated potassium (KCa3.1) channel blocker, senicapoc, would reduce cerebral edema and augment mean arterial pressure in the early post-resuscitation period.

**Method:**

Male Sprague-Dawley rats, aged 11–15 weeks, were utilized in the study. Rats were exposed to 8 min of asphyxial cardiac arrest. Shortly after resuscitation, rats were randomized to receive either vehicle or senicapoc (10 mg/kg) intravenously. The primary outcome was cerebral wet to dry weight ratio 4 h after resuscitation. Secondary outcomes included mean arterial pressure, cardiac output, norepinephrine dose, inflammatory cytokines and neuron specific enolase levels. Additionally, a sub-study was conducted to validate intravenous administration of senicapoc.

**Results:**

The sub-study revealed that senicapoc-treated rats maintained a significantly higher mean arterial pressure during administration of SKA-31 (a KCa3.1 channel opener).

The plasma concentration of senicapoc was 1060 ± 303 ng/ml 4 h after administration. Senicapoc did not reduce cerebral edema or augment mean arterial pressure 4 h after resuscitation. Likewise, cardiac function and norepinephrine dose did not vary between groups. Inflammatory cytokines and neuron specific enolase levels increased in both groups after resuscitation with no difference between groups. Senicapoc enhanced the PaO_2_/FiO_2_ ratio significantly 4 h after resuscitation.

**Conclusion:**

Senicapoc was successfully administered intravenously after resuscitation, but did not reduce cerebral edema or increase mean arterial pressure in the early post-resuscitation period.

## Highlights

• Senicapoc was successfully administered intravenously.
• Senicapoc did not reduce cerebral edema 4 h after cardiac arrest.
• Senicapoc did not increase mean arterial pressure within 4 h from resuscitation.

## Introduction

Neurological injury progresses over time following cardiac arrest and subsequent resuscitation.^1^, ^2^ The formation of cerebral edema, disturbed cerebral blood flow, cardiovascular dysfunction and an extensive inflammatory response are considered important contributing factors regarding the delayed cerebral injury.^3^, ^4^, ^5^, ^6^ Cerebral injury remains the most frequent cause of death and morbidity among cardiac arrest patients.^7^ Hence, there is a demand for novel treatment strategies to reduce risk of further neurological injury after cardiac arrest. The selective blocker of intermediate calcium-activated potassium (KCa3.1) channels, senicapoc,^8^ may represent such a novel treatment.

In vascular endothelial cells, activation of KCa3.1 channels cause vasodilation through the release of endothelial derived hyperpolarization factor (EDH).^8^, ^9^, ^10^ Results from our research group indicate that activation of cerebral endothelial KCa3.1 and small calcium-activated potassium (KCa2.x) channels yield an enhanced vasodilatory response in the early post-resuscitation period.^11^ Similarly, KCa3.1 and KCa2.x channel opening demonstrated enhanced vasodilation in mesenteric arteries after cardiac arrest,^12^ suggesting KCa3.1 channel activation as a mechanism of cardiovascular instability after cardiac arrest.

In the blood-brain barrier endothelial cells, evidence supports that KCa3.1 channels co-manage the regulation of ion and fluid transport across the blood-brain barrier.^13^ In the event of hypoxia, intracellular calcium increases, which activates the KCa3.1 channels. This entails a K^+^ efflux, which stimulates the Na^+^–K^+^–Cl^−^-cotransporter to drive chloride and water into the cerebral extracellular space.^14^

In immunological active cells, KCa3.1 channels may play a role in the regulation of cytokine production, migration and proliferation.^15^, ^16^, ^17^ This is supported by a wide range of studies reporting a diminished inflammatory response with KCa3.1 channel inhibition.^8^, ^18^, ^19^, ^20^, ^21^, ^22^

Therefore, we hypothesized that senicapoc improves cardiovascular function and reduces cerebral edema after cardiac arrest. The primary outcome was cerebral wet to dry weight ratio. Secondary outcomes were mean arterial pressure (MAP), cardiac output, norepinephrine dose, inflammatory cytokines and neuron specific enolase (NSE) levels.

## Experimental procedure

### Drug preparation

Senicapoc (MedChemExpress, Monmouth Junction, NJ, USA) was dissolved in 100% PEG-400 and stored at −18 °C. Shortly before administration, the stock solution was further diluted and finally consisted of 75% saline, 20% PEG-400 and 5% cremophor with a senicapoc concentration of 6.5 mg/ml. Naphtho[1,2-d]thiazol-2-ylamine (SKA-31) was dissolved in the same ratio of solvents. The vehicle solution consisted of the same solvents, but did not contain senicapoc.

### Ethical statement

The study was approved by the National Committee on Animal Research Ethics no. 2017-15-0201-01206 (Animal Experiments Inspectorate, Copenhagen, Denmark) and conducted in accordance with the “Guide for the Care and Use of Laboratory Animals”.^23^ The article and related results are reported in compliance with the ARRIVE guidelines.

### Housing and period of time

Male Sprague Daley rats (Taconic, Silkeborg, Denmark), aged 11–15 weeks, weighing 462 ± 78 g, were housed pairwise at standard room temperature (22−23 °C), humidity (45%), 12-h light/dark cycle and with free access to food and water. Data collection took place in September 2019–December 2019. All experiments were started in the morning between 8.00–10.00 am.

### Rat preparation

Anesthesia was induced by a subcutaneous injection of FFM mixture (fentanyl 0.0788 mg/ml, fluanisone 2.5 mg/ml, and midazolam 1.25 mg/ml) (2.4 ml/kg). A single dose of ketamine (100 mg/kg) was administered intraperitoneally to prevent pharyngeal reflexes 1 min before intubation. ^24^ Rats were orally intubated and ventilated with a tidal volume of 8 ml/kg. Supplemental oxygen and ventilation rate were adjusted to maintain PaO_2_ at 80−110 mmHg and PaCO_2_ at 35−45 mmHg. Temperature was kept at 36.5–37.5 °C by a feedback-controlled heating pad. The femoral artery and vein were catheterized for invasive blood pressure measurement, arterial sampling, fluid and drug administration. Saline (2 ml/kg/h) administration, maintenance of anesthesia, blood gas analysis and physiological monitoring were performed as previously described.^24^, ^25^

### Effect of senicapoc on blood pressure lowering

A separate sub-study was conducted to ensure appropriate concentration and administration of intravenous senicapoc. SKA-31 is an activator of KCa3.1 and KCa2.x channels, and causes a transient drop in blood pressure.^26^ We expected administration of the potent KCa3.1 channel blocker, senicapoc, to counteract the effect of SKA-31.

Rats, comparable to the ones used in the main study, were surgically prepared as described in section 2.4 and randomized to receive either vehicle or senicapoc (10 mg/kg) by drawing from an opaque envelope. The primary investigator was blinded to randomization and administration of drug(s). Rats were allowed to stabilize 1 h after senicapoc administration before they received an intravenous bolus of SKA-31 (3 mg/kg). SKA-31 administration was standardized using an infusion pump with a rate of 0.6 ml/min, which corresponded to an average infusion time of 35 s. Arterial gases were obtained at baseline (senicapoc administration), and after 50 and 90 min.

### Cardiac arrest and resuscitation

Asphyxial cardiac arrest was induced by turning off the ventilator preceded by rocuronium (2.4 mg/kg) administration in order to prevent spontaneous respiration. Cardiac arrest was defined as a MAP < 20 mmHg. After 8 min of cardiac arrest, epinephrine 0.01 mg/kg (every two minutes) was administered, while ventilation with 100% oxygen and chest compressions were initiated as previously described.^24^, ^25^ Return of spontaneous circulation (ROSC) was defined as a MAP > 40 mmHg. Resuscitation was discontinued if ROSC did not occur within 8 min.

Randomization was performed by drawing from an opaque envelope at the time of ROSC. The primary investigator was blinded to group assignment. Rats were randomized 1:1 into two groups (vehicle; n = 11 or senicapoc; n = 11) by an independent third person, who also prepared and administered senicapoc (10 mg/kg) or vehicle within 10 min from ROSC. The solution was infused at a rate of 0.2 ml/min corresponding to an average infusion time of 3 min and 33 s.

After ROSC, oxygen administration and ventilation were adjusted in agreement with arterial blood samples. If MAP dropped below 50 mmHg, norepinephrine (0.3 mg/ml) infusion was initiated at 0.24 ml/h and titrated to secure a MAP of 50−60 mmHg during the observation period.^27^, ^28^ Norepinephrine infusion was discontinued if MAP stabilized >60 mmHg. Saline infusion was adjusted in accordance with norepinephrine infusion to secure equal fluid administration. Rats were decapitated at the end of the observation period. Immediately after decapitation, cerebrum and lungs were extracted to measure wet weight. The corresponding dry weight was measured 7 days later. A figure illustrating the main study process is available in the supplementary material (eFig. 1).

### Echocardiography

Transthoracic 2D echocardiography was performed with a GE Healthcare Vivid S6 ultrasound system using an 11-MHz probe (12S-RS, GE Healthcare, Copenhagen, Denmark). Echocardiography was carried out at baseline, 2 and 4 h after ROSC as previously described.^29^ Additionally, right ventricular function was assessed through tricuspid annular plane systolic excursion (TAPSE) measurements. All dimensions were based on an average for three consecutive cardiac cycles.

Image analysis was performed in a randomized and blinded manner after data collection was concluded (EchoPac, GE Healthcare, Copenhagen, Denmark). The analysis was performed by the primary investigator and an independent third person. The mean difference and 95% limits of agreement were -54 ml/kg/min (-159; 55) for cardiac output, -0.06 ml (−0.17; 0.06) for stroke volume, −3.8% (−11.2; 3.6) for left ventricular ejection fraction, −7.6% (−22.3; 7.2) for left ventricular fractional shortening and −0.20 mm (−0.67; 0.27) for TAPSE.

### NSE, inflammatory cytokine measurements and senicapoc plasma concentration

Serum and plasma samples were collected at baseline and 4 h after ROSC. Samples were handled, centrifuged and analyzed as previously described.^29^ Based on previous studies,^12^, ^30^, ^31^ plasma concentration of specific inflammatory cytokines (IL-1α, IL-1β, IL-6, IL-10 and TNF-α) were measured with a multiplex assay (Bio-Rad Cytokine 5-plex Assay Kit, Hercules, CA, USA). The lower detection limits (pg/ml) were IL-1α (0.38), IL-1β (0.74), IL-6 (27), IL-10 (3.11) and TNF-α (12.34). Assessment of plasma senicapoc concentration was performed with sensitive liquid chromatography-tandem mass spectrometry.^32^ The lower detection limit was 0.1 μg/l. All blood samples were analyzed by personnel blinded to allocation.

### Statistics and sample size

Distribution was evaluated using QQ plots and histograms and log-transformed to obtain Gaussian distribution if needed. A non-parametric statistical test was applied if Gaussian distribution could not be achieved. Gaussian distributed data are presented as mean ± SD, whereas non-gaussian distributed data are reported as median with associated quartiles [Q25%; Q75%].

Temporal physiological measurements and echocardiography data were analyzed using repeated measurement ANOVA in order to assess whether there was a different development within and between groups over time. Additionally, echocardiography and physiological baseline and 4-h values were compared using an unpaired *t*-test. Fluid administration, blood sample measurements, wet to dry weight ratios and AUC data were also assessed between groups with an unpaired *t*-test.

For cytokines and NSE measurements, we performed a paired *t*-test from baseline to 4 h to evaluate the effect of cardiac arrest itself.

Two-tailed P-values <0.05 were considered statistically significant. Data analysis was carried out blinded. The estimated number of rats in each group was based on cerebral wet to dry weight ratios from pilot studies (n = 6; *α* = 0.05; β = 0.2; means ± SD: vehicle 4.717 ± 0.002 and senicapoc 4.660 ± 0.036). However, as the variation in the wet to dry weight ratio was larger than initially calculated, the number of rats was increased to 11 in each group.

All statistics were performed in GraphPad Prism (version 9.0.0, GraphPad Software Inc., CA, USA).

## Results

### Effect of senicapoc on blood pressure lowering

**Fig. 1:**
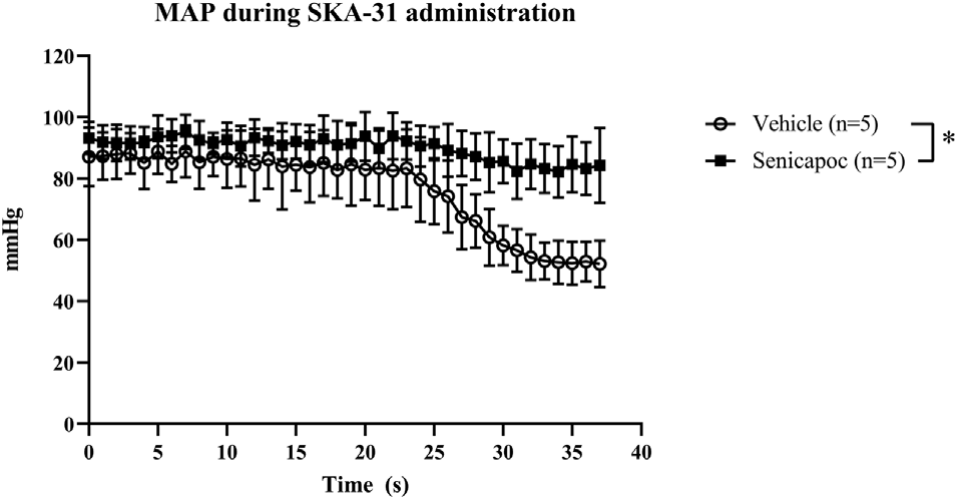
Mean arterial pressure (MAP) during intravenous infusion of SKA-31. Infusion pump is started at time 0. n = 5 per group. Data presented as mean ± SD. *=significant difference in AUC between vehicle and senicapoc-treated rats (p < 0.05).

Ten rats were utilized in the sub-study. Weight, infusion time and blood gas parameters were similar between groups at baseline, 50 min and 90 min (Supplementary material, eTable 1). Neither heart rate nor MAP differed between groups before SKA-31 administration. Intravenous SKA-31 administration caused a transient drop in MAP that was prevented in the senicapoc group (Fig. 1). Heart rate was not affected during the infusion of SKA-31, but decreased similarly in both groups shortly after SKA-31 administration (Supplementary material, eFig. 2).

### Rats exposed to cardiac arrest

Thirty-two rats were utilized in the cardiac arrest study. Ten rats did not obtain ROSC; hence twenty-two rats were randomized to receive either vehicle or senicapoc. Weight was comparable between groups (vehicle: 389 ± 26 g and senicapoc: 396 ± 26 g; p = 0.53).

### Physiological and cardiac arrest parameters

**Fig. 2:**
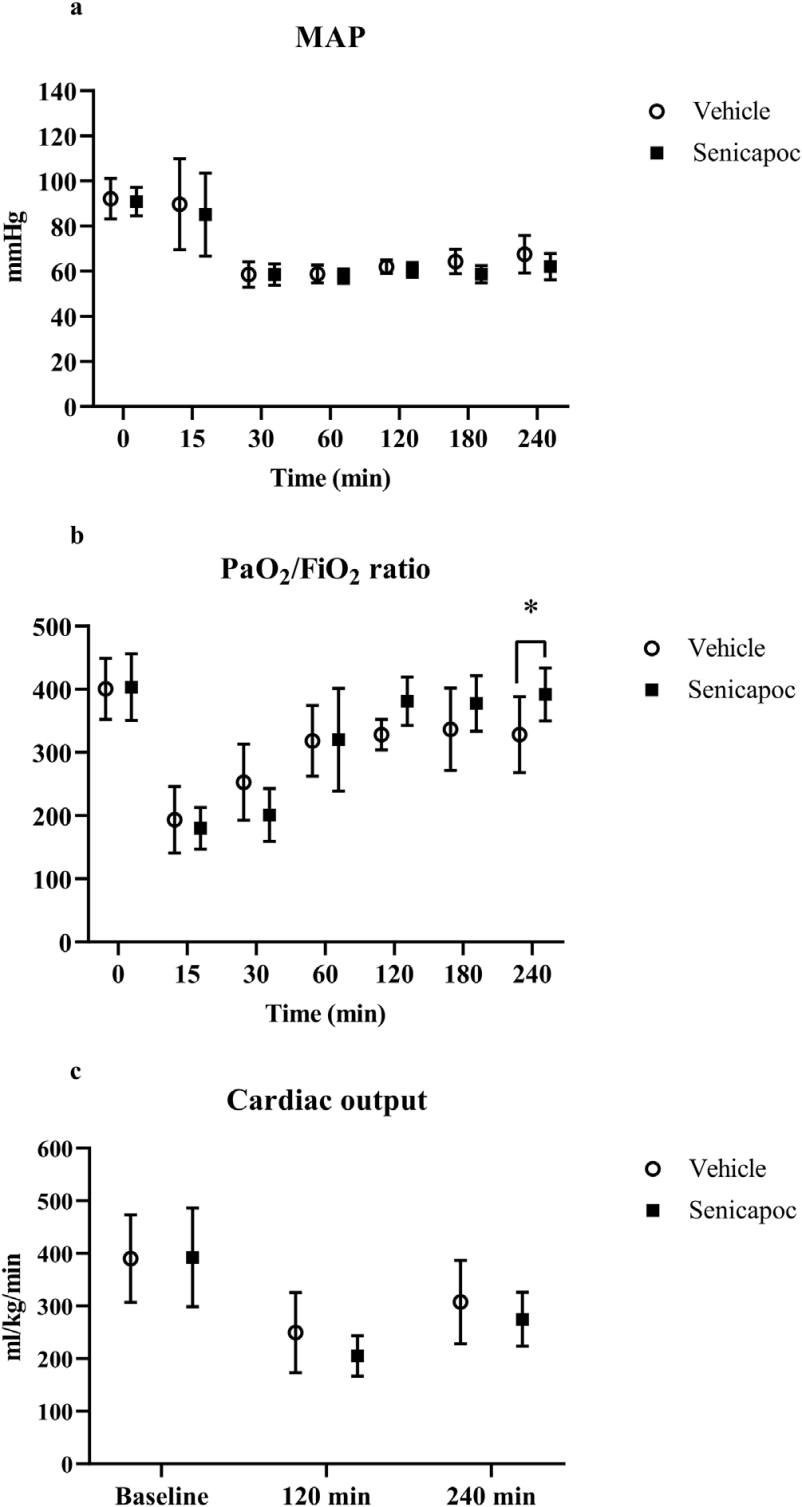
Development of mean arterial pressure (MAP), PaO_2_/FiO_2_ ratio and cardiac output over time. Data presented as mean ± SD. * = significant difference between vehicle and senicapoc-treated rats at 4 h (p < 0.05).

Physiological parameters were comparable between groups at baseline (Table 1 and Fig. 2). Likewise, time from asphyxia to cardiac arrest (vehicle: 74 ± 13 s and senicapoc: 73 ± 15 s; p = 0.9) and time from CPR to ROSC (vehicle: 126 ± 56 s and senicapoc: 118 ± 34 s; p = 0.67) were comparable between groups.

**Table 1:** Physiological parameters. n = 11 per group. Data presented as mean ± SD. HR = heart rate.

	Group	Baseline	15 min	30 min	1 h	2 h	3 h	4 h
HR (1/min)	Vehicle	389 ± 70	319 ± 40	294 ± 48	273 ± 39	281 ± 41	333 ± 48	363 ± 61#
	Senicapoc	395 ± 60	308 ± 43	274 ± 39	257 ± 27	249 ± 26	290 ± 46	325 ± 51#
PaCO_2_ (mmHg)	Vehicle	39 ± 4	41 ± 5	38 ± 3	36 ± 3	37 ± 3	38 ± 3	38 ± 3#
	Senicapoc	40 ± 3	42 ± 4	39 ± 4	36 ± 3	37 ± 2	37 ± 4	37 ± 4#
pH	Vehicle	7.44 ± 0.03	7.29 ± 0.07	7.33 ± 0.04	7.40 ± 0.02	7.40 ± 0.03	7.39 ± 0.03	7.39 ± 0.03#
	Senicapoc	7.44 ± 0.04	7.3 ± 0.05	7.32 ± 0.05	7.41 ± 0.05	7.41 ± 0.03	7.39 ± 0.05	7.38 ± 0.04#
PaO_2_ (mmHg)	Vehicle	107 ± 8	116 ± 19	105 ± 5	100 ± 10	101 ± 9	105 ± 9	104 ± 6#
	Senicapoc	110 ± 12	121 ± 28	108 ± 12	111 ± 12	104 ± 9	102 ± 6	105 ± 7#
Lactate (mmol/l)	Vehicle	1.0 ± 0.3	3.9 ± 0.8	3.2 ± 0.7	1.9 ± 0.5	1.2 ± 0.4	1.0 ± 0.3	0.9 ± 0.2#
	Senicapoc	0.9 ± 0.3	3.7 ± 0.7	2.9 ± 0.7	1.5 ± 0.4	1.1 ± 0.4	0.9 ± 0.2	0.8 ± 0.2#

# = significant difference over time within group (p < 0.05).

Repeated measurement ANOVA did not reveal any significant difference over time in any physiological parameters between the two groups. At 4 h, however, the PaO_2_/FiO_2_ ratio was significantly higher in the senicapoc group when compared to the vehicle group (Fig. 2b). Administration of norepinephrine in the observation period was comparable between the groups (vehicle: 1.2 [0.7; 1.5] ml/kg and senicapoc: 1.3 [1.0; 1.5] ml/kg; p = 0.70).

### Echocardiography

Cardiac output data is presented in Fig. 2c, while remaining echocardiography parameters are presented in Table 2. Apart from TAPSE, echocardiography measurements were comparable between groups at baseline. Repeated measurements ANOVA showed a significant decline in cardiac output, stroke volume, ejection fraction, fractional shortening and TAPSE after cardiac arrest; however, the changes were similar in both groups over time with no difference at 4 h.

**Table 2:** Echocardiography measurements at baseline, 2 h and 4 h. n = 11 per group. Ejection fraction (EF) and fractional shortening (FS) are calculated from the parasternal long axis projection. TAPSE = tricuspid annular plane systolic excursion. Data presented as mean ± SD.

	Group	Stroke volume (ml)	EF (%)	FS (%)	TAPSE (mm)
Baseline	Vehicle	0.40 ± 0.06	96 ± 4	71 ± 11	1.8 ± 0.2
	Senicapoc	0.39 ± 0.06	96 ± 4	72 ± 12	1.7 ± 0.1*
2 h	Vehicle	0.33 ± 0.06	92 ± 7	63 ± 14	1.5 ± 0.1
	Senicapoc	0.32 ± 0.05	87 ± 8	54 ± 14	1.7 ± 0.1
4 h	Vehicle	0.33 ± 0.03	93 ± 6	65 ± 15	1.7 ± 0.2
	Senicapoc	0.33 ± 0.05	90 ± 9	60 ± 16	1.7 ± 0.2

* = significant difference between vehicle and senicapoc-treated rats at baseline (p < 0.05).

### Wet to dry weight ratio

The cerebral wet to dry weight ratio was similar between the vehicle and senicapoc group (vehicle: 4.779 ± 0.069 and senicapoc: 4.780 ± 0.062, p = 0.98). Lung wet to dry weight ratio was likewise comparable between groups (vehicle: 4.732 ± 0.229 and senicapoc: 4.696 ± 0.157, p = 0.67).

### Blood sample measurements

**Fig. 3:**
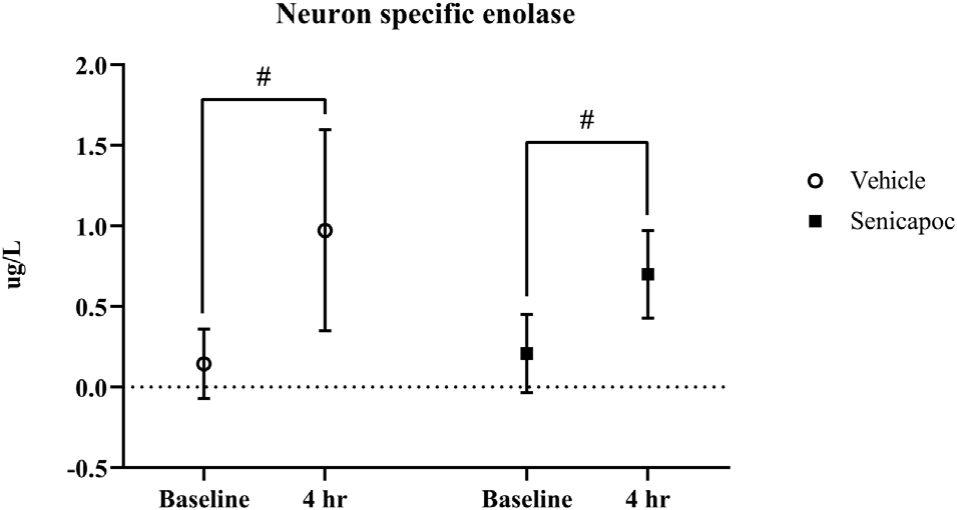
Neuron specific enolase levels at baseline and 4 h. n = 11 per group. Data presented as mean ± SD. # = significant difference from baseline to 4 h within group (p < 0.05).

NSE and inflammatory cytokine levels were similar in the experimental groups at baseline and 4 h after cardiac arrest (Fig. 3 and Table 3). NSE levels increased significantly in both groups after resuscitation. IL-6 and TNF-α also increased in both groups after resuscitation, but they only increased significantly from baseline to 4 h in the senicapoc group. The mean senicapoc plasma concentration was 1060 ± 303 ng/ml 4 h after administration.

**Table 3:** Plasma concentration of inflammatory cytokines at baseline and 4 h. n = 11 per group. Data presented as mean ± SD.

	Group	IL-1α (pg/ml)	IL-1β (pg/ml)	IL-6 (pg/ml)	IL-10 (pg/ml)	TNF-α (pg/ml)
Baseline	Vehicle	1 ± 2	1 ± 2	0 ± 0	33 ± 15	23 ± 15
	Senicapoc	1 ± 1	2 ± 5	0 ± 0	37 ± 17	17 ± 12
4 h	Vehicle	1 ± 1	2 ± 2	12 ± 23	28 ± 14	33 ± 15
	Senicapoc	0 ± 0	5 ± 4	15 ± 22#	32 ± 13	37 ± 22#

# = significant difference from baseline to 4 h within group (p < 0.05).

## Discussion

We investigated the effect of senicapoc on cardiovascular function and cerebral edema formation in rats after resuscitation from cardiac arrest. We are, to our knowledge, the first to succeed in administering senicapoc intravenously, which is supported by satisfactory senicapoc plasma concentrations and senicapoc’s ability to counteract the effect of SKA-31. Senicapoc did not alter MAP, cerebral edema, norepinephrine dose, cardiac output, inflammatory cytokine levels or NSE levels 4 h after cardiac arrest. The PaO_2_/FiO_2_ ratio, on the other hand, was significantly higher in senicapoc-treated rats when compared to vehicle rats 4 h after cardiac arrest.

Senicapoc has primarily been investigated in connection with non-acute diseases, where it has been administered orally.^33^, ^34^, ^35^, ^36^, ^37^ The acute nature of cardiac arrest, however, entails an efficient and fast way of administering drugs; i.e., intravenously. Our intravenous approach and dosing were validated through senicapoc plasma concentration measurements and an in vivo sub-study. Senicapoc plasma concentrations reached a mean of 3278 ± 936 nM (1060 ± 303 ng/ml) in senicapoc-treated rats 4 h after administration, which is above the half maximal inhibitory concentration of 11 nM.^8^ In the sub-study, SKA-31 caused a transient drop in blood pressure unrelated to heart rate, which is in line with several other studies.^38^, ^39^, ^40^ The transient drop was prevented in the senicapoc group indicating an efficient blockage of KCa3.1 channels. These findings show that senicapoc exerted maximum blockade of the KCa3.1 channels.

Despite senicapoc’s ability to maintain a higher MAP in the sub-study, we did not find any effect of senicapoc on MAP or norepinephrine requirement after resuscitation. This finding was contrary to our hypothesis, where senicapoc would increase MAP or decrease the need for norepinephrine. This was based on (1) mesenteric arteries contribute substantially to the regulation of peripheral resistance and thus MAP,^41^ (2) rat mesenteric arteries display enhanced EDH-vasodilation in vitro after cardiac arrest,^12^ and (3) MAP is elevated in KCa3.1 channel knockout mice.^42^ Our findings, however, indicate that other mechanisms are likely to be the main contributors to the systemic hypotension observed in the early post-resuscitation period. One such mechanism may be the inflammatory response that arise as a consequence of the ischemia-reperfusion injury following resuscitation.^6^, ^43^ Elevated levels of cytokines and endothelial derived biomarkers is a frequent finding after cardiac arrest,^44^, ^45^, ^46^ which is in line with our data demonstrating elevated levels of IL-6 and TNF-α 4 h after resuscitation. The inflammatory response impairs endothelial function and augment vascular permeability, which entails abnormal vasoregulation and systemic hypotension.^6^, ^47^ However, it is worth noting that the impact of vascular dysfunction has been proposed to increase gradually >8 h after resuscitation, whereas myocardial dysfunction seem to be the main component of systemic hypotension earlier in the post-resuscitation period.^45^, ^48^ This is supported by our echocardiography data, where cardiac output is reduced at hours 2 approaching normal levels at 4 h. These observations advocate that a potential vascular effect of senicapoc on MAP may show later than 4 h after resuscitation. Here, it should be noted though that blockage of KCa3.1 channels has been shown to improve cardiac function albeit it was in a myocardial infarction model with long-term treatment.^49^

Contrary to our hypothesis, we found no effect of senicapoc on cerebral injury between vehicle and senicapoc-treated rats. NSE levels increased significantly in both groups after cardiac arrest, indicating cerebral injury in line with previous studies performed by our research group.^11^, ^30^ The current results are in contrast to results from stroke studies in rats and mice, where KCa3.1 channel blockage reduced both infarct size and neuroinflammation while also improving neurological outcome. These studies, however, had a considerably longer follow-up period of 7 days as compared to our 4 h.^14^, ^22^

KCa3.1 channel inhibition has also been shown to reduce cerebral edema 4 h after stroke and 24 h after traumatic brain injury in rats.^13^, ^50^ This is of particular interest as cerebral edema has been linked to the progression of neurological injury after cardiac arrest.^5^ Contrary to our pilot studies, we did not detect any reduction in cerebral edema between groups. The formation of cerebral edema is often divided into two associated phases; i.e. the ionic and vasogenic phase.^51^ The vasogenic phase, considered to be the most influential, seem to arise several hours after resuscitation and may even persist for days.^52^ Therefore, a potential difference in edema formation may have been too limited for us to detect with a wet to dry weight ratio method only 4 h after cardiac arrest. That KCa3.1 channels may play an important role in the regulation of fluid transport into the interstitial tissue, is further highlighted by a mouse model of lung injury established by our research group. Here, senicapoc was capable of reducing lung edema and increase the PaO_2_/FiO_2_ ratio when compared to vehicle mice.^53^ Interestingly, the current study also found the PaO_2_/FiO_2_ ratio to be significantly enhanced in senicapoc-treated rats 4 h after cardiac arrest. It has to be stressed, though, that this was a secondary finding.

### Limitations

A longer observation period would have strengthened the current study. However, an observation period of 4 h was chosen due to (1) studies by our research group indicating significantly enhanced endothelial KCa3.1 channel signaling 2 and 4 h after cardiac arrest,^11^, ^12^ and (2) KCa3.1 channel inhibition showing a significant reduction in cerebral edema 4 h after stroke in rats.^13^ In the latter study, MRI was utilized to detect the early effect of KCa3.1 channel inhibition. MRI has also been applied to detect cerebral edema in a cardiac arrest rat study,^54^ and is indisputable a more accurate way of measuring cerebral edema formation. An MRI assessment would have strengthened our results and enhanced the translational impact of our study, but unstable hemodynamics make MRI challenging in the early post-resuscitation period. Yet, cerebral wet to dry weight has previously been used to evaluate the effect of hypothermia as early as 1 h after cardiac arrest in rats.^55^ Moreover, it should be noted that sham-operated rats had a lower wet to dry weight ratio (4.547 ± 0.064, n = 3) when compared to cardiac arrest rats with our setup (unpublished results).

## Conclusion

Senicapoc did not reduce the formation of cerebral edema 4 h after cardiac arrest. Nor was norepinephrine dose reduced or MAP higher in senicapoc-treated rats when compared to vehicle. This study is the first to report successful intravenous administration of senicapoc. Further studies are required to confirm the higher PaO_2_/FiO_2_ ratio in senicapoc-treated rats.

## CRediT authorship contribution statement

**Frederik Boe Hansen:** Study concept and design, Acquisition of data, Analysis and interpretation of data, Drafting of the manuscript, Statistical analysis, Obtained funding. **Niels Secher:** Study concept and design, Analysis and interpretation of data, Critical revision of the manuscript for important intellectual content, Study supervision(s). **Thomas Mattson:** Analysis and interpretation of data, Critical revision of the manuscript for important intellectual content, Statistical analysis. **Bo Løfgren:** Study concept and design, Analysis and interpretation of data, Critical revision of the manuscript for important intellectual content, Study supervision(s). **Ulf Simonsen:** Study concept and design, Analysis and interpretation of data, Drafting of the manuscript, Critical revision of the manuscript for important intellectual content, Administrative, technical, and material support, Study supervision(s). **Asger Granfeldt:** Study concept and design, Analysis and interpretation of data, Drafting of the manuscript, Critical revision of the manuscript for important intellectual content, Statistical analysis, Administrative, technical, and material support, Study supervision(s).

All authors had full access to all the data and take responsibility for the integrity of the data and the accuracy of the data analysis.

## Funding

This work was supported by the King Christian X’s Foundation [grant no. not applicable]; the 10.13039/501100008249Frimodt-Heineke Foundation [grant no. not applicable]; and the A. P. Moeller Foundation [grant no. not applicable].

## Conflicts of interests

Granfeldt and Simonsen are inventors on a patent owned by Aarhus University claiming the use of senicapoc for ARDS caused by COVID-19. None of the grant organizations or Aarhus University, apart from the authors, have had an influence on the content of the article. The other authors report no conflicts.
