# Supplementary material for: Effect of the KCa3.1 blocker, senicapoc, on cerebral edema and cardiovascular function after cardiac arrest — A randomized experimental rat study
Source: Resusc Plus. 2021 Apr 2;6:100111. doi: 10.1016/j.resplu.2021.100111 (PMC8244250; doi:10.1016/j.resplu.2021.100111)
Supplement: Supplementary file 1 [file mmc1.docx]

Supplementary material

*Effect of the KCa3.1 blocker, senicapoc, on cerebral edema and cardiovascular function after cardiac arrest – a randomized experimental rat study*

**
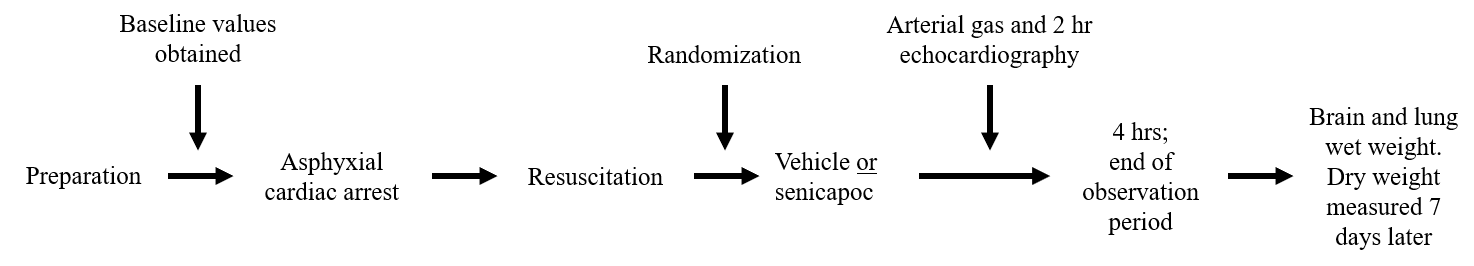
**

**eFigure 1:** Timeline for the main study**.**

**Additional sub-study information**

Weight: vehicle 459±74 g and senicapoc 466±90 g, p=0.91

Infusion time: vehicle 34±6 s and senicapoc 34±7 s, p=0.96

|  | **Group** | **Baseline** | **50 min** | **90 min** |
| --- | --- | --- | --- | --- |
| **PaO2  (mmHg)** | Vehicle | 140±26 | 128±21 | 122±9 |
|  | Senicapoc | 117±29 | 123±16 | 129±10 |
| **PaCO_2_  (mmHg)** | Vehicle | 36±3 | 35±2 | 36±2 |
|  | Senicapoc | 39±3 | 36±2 | 36±3 |
| **Saturation (%)** | Vehicle | 100±0 | 100±0 | 100±0 |
|  | Senicapoc | 97±4 | 100±1 | 100±0 |
| **pH** | Vehicle | 7.50±0.04 | 7.51±0.02 | 7.60±0.21 |
|  | Senicapoc | 7.46±0.03 | 7.49±0.02 | 7.50±0.03 |
| **Base excess (mmol/l)** | Vehicle | 4.6±1.5 | 5.0±1.4 | 5.0±1.2 |
|  | Senicapoc | 4.0±1.2 | 4.8±1.2 | 5.0±2.1 |
| **Lactate (mmol/l)** | Vehicle | 0.9±0.2 | 1.1±0.3 | 0.8±0.3 |
|  | Senicapoc | 0.9±0.2 | 1.0±0.3 | 0.8±0.2 |

**eTable 1:** Blood gas parameters. n=5 per group. Data presented as mean±SD.

**eFigure 2:** Heart rate measurements in vehicle and senicapoc-treated rats at the time of SKA-31 administration.
